# Randomized Cross Over Study Assessing the Efficacy of Non-invasive Stimulation of the Vagus Nerve in Patients With Axial Spondyloarthritis Resistant to Biotherapies: The ESNV-SPA Study Protocol

**DOI:** 10.3389/fnhum.2021.679775

**Published:** 2021-06-30

**Authors:** Eric Azabou, Guillaume Bao, Félicie Costantino, Madalina Jacota, Chanez Lazizi, Lionelle Nkam, Martin Rottman, Anne-Laure Roux, Sylvain Chevallier, Lamiae Grimaldi, Maxime Breban

**Affiliations:** ^1^Clinical Neurophysiology and Neuromodulation Unit, Department of Physiology, Raymond Poincaré Hospital, Assistance Publique-Hôpitaux de Paris (AP-HP), Paris, France; ^2^Laboratory of Infection and Inflammation (2I)–Inserm UMR 1173, University of Versailles Saint-Quentin en Yvelines (UVSQ), Paris-Saclay University, Paris, France; ^3^Rheumatology Department, AP-HP, Ambroise Paré Hospital, AP-HP, Boulogne-Billancourt, France; ^4^Laboratory of Excellence Inflamex, Paris Descartes University, Sorbonne Paris Cité, Paris, France; ^5^Clinical Research Unit, Ambroise Paré Hospital, Assistance Publique-Hôpitaux de Paris (AP-HP), Boulogne-Billancourt, France, University of Versailles Saint-Quentin en Yvelines, Paris-Saclay University, Paris, France; ^6^Microbiology Laboratory, Raymond Poincaré Hospital, AP-HP Paris Saclay University, Paris, France; ^7^Versailles Engineering Systems Laboratory (LISV), University of Versailles Saint Quentin en Yvelines (UVSQ), Vélizy, France

**Keywords:** axial spondyloarthritis, medical device, vagus nerve, trial, protocol, neuromodulation

## Abstract

Axial spondyloarthritis (SpA), is a major cause of chronic pain and disability that profoundly alters the quality of life of patients. Nearly half of patients with SpA usually develop drug resistance. Non-pharmacological treatments targeting inflammation are an attractive alternative to drug administration. Vagus nerve stimulation (VNS), by promoting a cholinergic anti-inflammatory reflex holds promise for treating inflammatory disease. Inflammatory reflex signaling, which is enhanced by electrically stimulating the vagus nerve, significantly reduces cytokine production and attenuates disease severity in animal models of endotoxemia, sepsis, colitis, and other preclinical models of inflammatory diseases. It has been proposed that vagal efferent fibers release acetylcholine (Ach), which can interact with α7-subunit-containing nicotinic receptors expressed by tissue macrophages and other immune cells to rapidly inhibit the synthesis/release of pro-inflammatory cytokines such as TNFα, IL-1β, IL-6, and IL-18. External vagal nerve stimulation devices are now available that do not require surgery nor implantation to non-invasively stimulate the vagal nerve. This double-blind randomized cross-over clinical trial aims to study the change in SpA disease activity, according to Assessment in Ankylosing Spondylitis 20 (ASAS20) definition, after 12 weeks of non-invasive VNS treatment vs. non-specific dummy stimulation (control group). One hundred and twenty adult patients with drug resistant SpA, meeting the ASAS classification criteria, will be included in the study. Patients will be randomized into two parallel groups according to a cross over design: either active VNS for 12 weeks, then dummy stimulation for 12 weeks, or dummy stimulation for 12 weeks, then active VNS for 12 weeks. The two stimulation periods will be separated by a 4 weeks wash-out period. A transcutaneous auricular vagus nerve stimulator Tens Eco Plus SCHWA MEDICO^TM^ France will be used in this study. The active VNS stimulation will be applied in the cymba conchae of the left ear upon the auricular branch of the vagus nerve, using low intensity (2–5 mA), once à week, during 1 h. Dummy stimulation will be performed under the same conditions and parameters as active VNS stimulation, but at an irrelevant anatomical site: the left ear lobule. This multicenter study was registered on ClinicalTrials.gov: NCT04286373.

## Introduction

Axial spondyloarthritis (SpA) is a chronic inflammatory disease primarily affecting the sacroiliac and spinal joints that usually begins in young adults and is a major cause of chronic pain and disability that profoundly alter the quality of life of patients (Taurog et al., 2016). Its prevalence is estimated at 0.4% of the French adult population (Costantino et al., 2015). Development of anti-tumor necrosis factor (TNF) and anti-interleukin (IL)-17 biotherapies have improved the management of these patients (Braun et al., 2002). However only half of the patients respond to these treatments and many of them are only partially relieved. Moreover, these treatments are expensive and can lead to serious side effects (Braun and Sieper, 2002; Wendling and Prati, 2011; Wendling et al., 2018). Electrical stimulation of the vagus nerve (VNS) recently emerged as an alternative to effectively attenuate peripheral inflammation in a variety of pathological conditions with few side effects (Borovikova et al., 2000; Yamakawa et al., 2013; Koopman et al., 2016). VNS which has been used for decades as a treatment of drug-resistant epilepsy and depression (O’Reardon et al., 2006) is known to generate a cholinergic anti-inflammatory reflex (Huston and Tracey, 2011; Cheng et al., 2014). There is experimental evidence for the role of the vagus nerve in regulating a number of distinct important physiological pathways including cerebral blood flow (CBF), melanocortin’s’ production, inflammation, glutamate excitotoxicity, norepinephrine and neurotrophic processes (Cai et al., 2014). Indeed, the vagus nerve regulates numerous central and peripheral key processes through afferent and efferent fibers (Berthoud and Neuhuber, 2000). The ascending afferent vagal nerve fibers can inform the central nervous system of the presence of peripheral inflammation (Maier et al., 1998). Anti-inflammatory reflex signaling, which is enhanced by electrically stimulating the vagus nerve, significantly reduces cytokine production and attenuates disease severity in animal models of endotoxemia (Mihaylova et al., 2012; Zhao et al., 2012), sepsis (Li et al., 2015), colitis (Meroni et al., 2018, 2019) and other preclinical models of inflammatory diseases (Li et al., 2015; Johnson et al., 2016; Ren et al., 2018; Huffman et al., 2019). The cholinergic anti-inflammatory pathways (CAP) were described by the group of KJ Tracey (Borovikova et al., 2000; Tracey, 2002; Pavlov and Tracey, 2012). Modulating the activity of the CAP has become an important therapeutic strategy for the management of inflammatory diseases (Huston, 2012; Zi et al., 2020; Wu et al., 2021). The CAP pathways innervate the spleen through the efferent vagus nerve and the splenic nerve relay, and act on macrophages by converting adrenergic stimulation into a cholinergic signal by the T cells of the spleen, which produce an anti-inflammatory effect (Hu et al., 2021).

It has been shown that vagal efferent fibers release acetylcholine (Ach), which can interact with α7-subunit-containing nicotinic receptors expressed on tissue macrophages and other immune cells to rapidly inhibit the synthesis/release of pro-inflammatory cytokines such as TNFα, IL-1β, IL-6, and IL- 18 (Borovikova et al., 2000; Rosas-Ballina et al., 2008).

The anti-inflammatory properties of VNS have been extensively confirmed under experimental conditions in inflammatory pathologies like arthritis, stroke, cardiovascular diseases and lupus (Leib et al., 2013; Jiang et al., 2014; Levine et al., 2014). These findings give rise to great expectations on its use to ameliorate neuro-inflammation and to contribute to the control of the progression of infectious and inflammatory diseases (Liu et al., 2014; Phillips et al., 2014; Steardo et al., 2015). VNS has been proven for decreasing inflammation and reducing symptoms in patients with inflammatory bowel disease which is associated to extraintestinal manifestations such as rheumatologic manifestations and TNF-alpha as a key cytokine in both diseases (Pellissier et al., 2014; Marshall et al., 2015; Bonaz et al., 2017; Fournier et al., 2018, 2020). It has also recently been successfully evaluated in rheumatoid arthritis (RA), in patients who had failed multiple lines of anti-TNF biotherapy, with a very good tolerance and a sustained decrease of disease activity equivalent to what is observed with the most effective pharmacological treatments (Koopman et al., 2016). On the other hand, VNS modulates nociception (Randich and Gebhart, 1992). It has been demonstrated that VNS attenuates pain via immunomodulation and neuro-immune interactions involving specialized proresolving mediators such as resolvins, protectins, maresins, and lipoxins (Moisset et al., 2020; Tao et al., 2020). VNS indeed produce anti-nociceptive effects through regulation of neuroinflammation in peripheral nerves, dorsal root ganglia/trigeminal ganglia, and spinal cord/brain-through neuro-immune interactions (Lange et al., 2011; Gaul et al., 2016; Silberstein et al., 2016). These findings suggest that VNS could be a promising adjunct therapy targeting inflammatory pathways in SpA patients. This hypothesis deserves to be investigated. Implanted VNS devices have been used in humans for decades to treat refractory partial-onset seizures and severe recurrent refractory depression with confirmed safety, efficacy and only mild to moderate side effects that are predictable and shown to improve over time (Ben-Menachem, 2001; Morris et al., 2013; Ryvlin et al., 2014). Non-invasive VNS devices are now available and expected to reduce the invasiveness of the procedure and increase its acceptability (Frangos et al., 2015; Fang et al., 2017).

## Main Objective of the Research

The primary objective of this study is to evaluate the change in SpA disease activity, according to ASsessment in AS International Working Group criteria for 20% improvement criteria ASAS20 (Anderson et al., 2001) after 12 weeks of VNS treatment in comparison with dummy stimulation period.

### Secondary Objectives

The secondary objectives of the study are to evaluate differences in disease evolution between the active and dummy stimulation periods of treatment, on the following parameters:

1. Change in disease activity according to “ASAS40” criteria (Sieper et al., 2009).
2. Obtaining a partial remission according to the ASAS definition.
3. Change in Bath Ankylosing Spondylitis Functional Index (BASFI).
4. Change in C-reactive protein (CRP) serum level and erythrocytes sedimentation rate (ESR).
5. Change in Ankylosing Spondylitis Disease Activity Score (ASDAS)_CRP and ASDAS_ESR.
6. Difference in levels of circulating cytokines: IL-6, IL-23, IL-17, IL-33, and of matrix metallopeptidases (MMP3-8-9).
7. Change in quality of life: assessment according to the following indexes: SF-36, AS Quality of Life (ASQOL) (Doward et al., 2003).
8. Change in Health Index of patient with SpA (ASAS HI) and of the Productivity at Work Index (WPI) (Kiltz et al., 2015).
9. Change in fatigue (Bath Ankylosing Spondylitis Disease Index (BASDAI) 1st question) and global pain.
10. Change in Anxiety and Depression Assessment (HAD) (Lepine et al., 1985).
11. Change in Bath Ankylosing Spondylitis Metrology Index BASMI (Jones et al., 1995).
12 Change in no-steroidal anti-inflammatory drug (NSAID) intake score (Dougados et al., 2011).

## Methods and Analysis

### Participants

The current article followed the SPIRIT guidelines^1^ and checklist. This randomized crossover placebo-controlled double blinded multicenter clinical trial will be conducted in rheumatology departments from 16 public hospitals in France. The trial has been approved by the Ethics Committee “Comité de protection des personnes du Sud-Ouest et Outre-Mer 4” (N°IRB: IORG0009855) on October 2nd 2020. The study was registered at www.clinicaltrials.gov (NCT04286373) prior to enrollment, and is part of the SMART-VNS^(TM)^ project, aimed at promoting research, development and technological innovation in the field of vagal neuromodulation therapy. It will be performed according to the declaration of Helsinki. Informed consent will be obtained from all participant for their participation in the study. One hundred and twenty adult SpA patients, fulfilling the ASAS classification criteria for axial SpA (Rudwaleit et al., 2009) and followed for at least 1 year, insufficiently relieved despite optimal drug management, including at least two lines of biotherapies tested for at least 6 months, will be enrolled in this study.

## Eligibility Criteria

### Inclusion Criteria

1. Adult patient (18–90 years old) meeting the ASAS classification criteria fo axial SpA, followed for at least 1 year, with radiographic evidence of advanced sacro-illitis, according to modified New-York criteria (van der Linden et al., 1984) (ankylosing spondylitis) or not.
2. Patient suffering of active SpA, with or without treatment, having a total BASDAI score ≥4 (0–10) at baseline and a score of global pain ≥4 (0–10).
3. Patient insufficiently relieved despite optimal drug management for at least 6 months, including at least 2 different NSAIDs at the maximum tolerated dose for at least 3 months (or less in case of intolerance) and at least two lines of biotherapies, or discontinued SpA treatments due to intolerance, contraindication.

### Exclusion Criteria

1. Patient under guardianship.
2. Cardiac arrhythmia.
3. Patients with Cochlear implant.
4. Patients with heart implant.
5. Asthmatic patient.
6. Refusal to participate in the study or to sign the informed consent.
7. Pregnant or breastfeeding woman.
8. No affiliation to a social security scheme.
9. Previous VNS treatment.
10. Incapacity to attend the weekly appointment during the study period.

In case of skin lesion of the left ear, recruitment will be delayed until the lesion is healed.

## Procedures

### Recruitment Procedure

This multi-center study will be conducted in Rheumatology departments from 16 Hospitals in France. The expected duration of the enrollment period is 12 months.

The screening visit will take place during a routine medical follow-up consultation in rheumatology.

The investigating physician will verify the inclusion and exclusion criteria. Eligible patients will be informed about the objectives and constraints of the study. Information note and consent form will be delivered.

After a reflection period of 1–30 days, if the patient gives his/her consent to participate to the research, patient and investigating physician will write their names on and sign the consent form at the inclusion visit. A copy of this form will be given to the patient, a copy will be kept by the investigating physician and a copy will be kept by the Sponsor of the study.

After obtaining the informed consent, the investigating physician will proceed to his/her enrollment in the study.

The patients who gave their written consent must be included within a month following the screening visit. The investigating physician will collect systematically the following data at the time of inclusion:

1. Demographic: date of birth, sex.
2. Main medical or surgical histories,
3. Date of diagnosis of SpA,
4. Previous lines of treatment received for SpA, including biotherapies, and their result,
5. Complementary examination results: HLA-B27 genotype, sacro-iliac joints scoring on pelvic radiograph (mandatory) and MRI (if available), as well as baseline data on SpA activity status and medication use.

Clinical and biological exams will be performed:

1. Weight and height.
2. Temperature, cardiac frequency, blood pressure.
3. Electrocardiogram.
4. Blood sample for assessment of levels of circulating cytokines: IL-6, IL-23, IL-17, IL-33, and of MMP3-8-9.

### Randomization

In this randomized 2^∗^2 cross-over trial, included patients will be randomized at inclusion in two groups differing by the sequence in which the treatments are to be administered: Group A: VNS active for 12 weeks, then dummy stimulation for 12 weeks; and Group B: dummy stimulation for 12 weeks then VNS active for 12 weeks. In order to maintain the blind, investigators administering the stimulation will be different from those evaluating the patients, and the latter will be blinded to the treatment administered. A centralized online randomization process will be performed and managed by the Entity responsible for monitoring the study.

Since the sites of stimulation on the left ear differ between the active and dummy periods, the patients might perceive differences between the sessions, but they will not be able to distinguish which sessions involve active VNS. Indeed, we consider that the few minor side effects that could be more frequent during the active VNS period are non-specific, rare and of little clinical importance and will not allow patients or physicians involved in endpoints assessment to identify the randomization group.

After randomization, only the investigators who apply VNS stimulation (active or dummy) will have access to the result of the randomization arm and will adapt the mode of application of the device. The staff involved in the stimulation sessions will communicate neither the type of session nor the randomization arm to the patients. Thus, patients will go to stimulation sessions without knowing whether it will be active or dummy stimulation. The physician in charge of evaluations will only receive the message that the patient has been randomized (without the result).

### Intervention and Device

Patients randomized in group A will receive active VNS stimulation once a week for 12 weeks starting from the first week after inclusion, followed by dummy stimulation for 12 weeks.

Patients randomized to group B will receive the reverse sequence: weekly dummy stimulation for 12 weeks, followed by 12 weeks of active VNS. The two stimulation periods will be separated by a 4-weeks (± 1 week) wash-out period. Sessions are not to be performed in case of skin lesions of the left ear or other acute medical conditions preventing the patient from attending the VNS sessions.

A transcutaneous vagus nerve stimulator Tens Eco Plus SCHWA MEDICO^TM^, France, will be used for this study. The active VNS stimulation will be applied in the hollow of the left outer ear on the auricular branch of the vagus nerve (cymba conchae), during a 1hour session of stimulation per week, at a weak intensity value (between 2 and 5 mA) and 2 Hz frequency. Stimulation modalities were determined according to preliminary observation performed in a small group of refractory SpA patients that received VNS for pain treatment (Azabou E. and Breban M., unpublished data). Dummy stimulation stimulation will be performed under the same conditions and parameters as active VNS stimulation, but at an irrelevant site: the left ear lobule, according to previously published methods (Frangos et al., 2015; Fang et al., 2017). Continuous EKG signal will be monitored and recorded during all the VNS sessions in order to analyze and scope cardiac rhythm variability and vagal tone.

### Follow-up Visits

**FIGURE 1:**
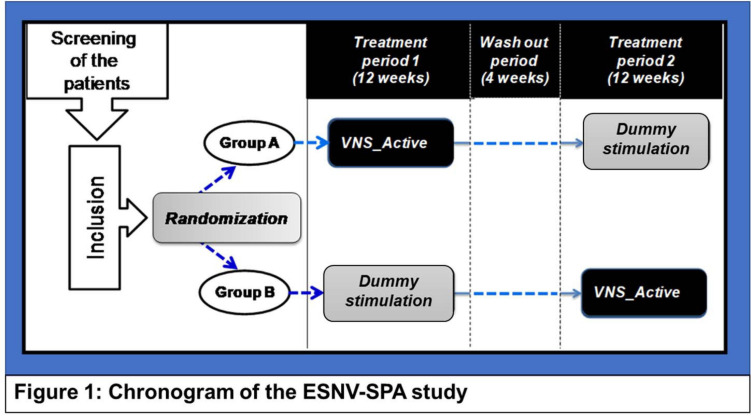
Chronogram of the ESNV-SPA study. Patients randomized in group A will receive active VNS stimulation once a week for 12 weeks starting from the first week after inclusion: Treatment period 1 (TP-1), followed by dummy stimulation for 12 weeks: Treatment period 2 (TP-2). Patients randomized to group B will receive the reverse sequence: weekly dummy stimulation for 12 weeks as TP-1, followed by 12 weeks of active VNS as TP-2. The two stimulation periods will be separated by a 4-weeks wash-out period.

During the first and the second treatment periods of 12 consecutive weekly stimulations, a weekly visit of each patient at the investigator site will be needed. These two periods will be separated by a 1-month washout period (see Table 1 and Figure 1).

**TABLE 1:** Organization of the ESNV-SPA study.

Actions	Screening visit (D0)	Baseline visit (D0 ± 30 days)	Treatment period 1 (TP1 = 12 weeks)	Wash out period (4weeks)	Treatment period 2 (TP2 = 12 weeks)	1 week after TP2
				1 week after TP1		
Verification of inclusion and exclusion criteria	X					
Information	X					
Informed consent	X	X				
Randomization		X				
Medical history	X					
Clinical examination including BASMI*	X	X	X	X	X	X
EKG		X	X		X	
Questionnaires**	X	X	X	X	X	X
Weekly Stimulation			X		X	
Blood samples*** (Biochemistry and cytokine assay)		X	X	X	X	X
Adverse events			X		X	

*Clinical examination to collect BASMI variables will be performed at the time of baseline visit, the first week of each treatment period and at the first week following each treatment period: Baseline, TP1_Week-1, Post-TP1_Week-1; TP2_Week-1, and Post-TP2_Week-1.

Questionnaires: The questionnaires listed in the “study endpoints” will be filled in at each weekly visit during each treatment period, but also at the first week following each treatment period: TP1_Week-1, TP1_Week-2, TP1_Week-3, TP1_Week-4, TP1_Week-5, TP1_Week-6, TP1_Week-7, TP1_Week-8, TP1_Week-9, TP1_Week-10, TP1_Week-11, TP1_Week-12, and Post-TP1_Week-1; followed by TP2_Week-1, TP2_Week-2, TP2_Week-3, TP2_Week-4, TP2_Week-5, TP2_Week-6, TP2_Week-7, TP2_Week-8, TP2_Week-8, TP2_Week-10, TP2_Week-11, TP2_Week-12, and Post-TP2_Week-1. **A total of 26 times of data collection.

Biological samples: Biological tests listed in the “study endpoints” section will be performed by blood sampling on a monthly basis: TP1_Week-1; TP1_Week-5; TP1_Week-9; and Post-TP1_Week-1; and TP2_Week-1; TP2_Week-5; TP2_Week-9; and Post-TP2_Week-1. ***A total of 8 times of data collection.

The expected length of participation for each patient is 8 months.

Twenty-six visits are planned during the protocol, according to the following schedule:

– Treatment period 1 (TP1): The duration of the first treatment period is 12 weeks. The patients will receive a weekly treatment from the first week of TP1 (TP1_Week-1) to the 12th week (TP1_Week-12).
– Washout period (WOP): The TP1 will be followed by a 4 weeks washout period (WOP).
– Treatment period 2 (TP2): The second treatment period duration is 12 weeks, and will start after the 4 weeks’ WOP. The patients will then receive a weekly treatment from the first week (TP2_Week-1) to the 12th week of the TP2 (TP2_Week-12).

### Data Collection at Endpoints

Clinical examination including BASMI assessment: Clinical examination to collect BASMI variables will be performed at the time of baseline visit, the first week of each treatment period and at the first week following each treatment period: Baseline, TP1_Week-1, Post-TP1_Week-1; TP2_Week-1, and Post-TP2_Week-1.

Questionnaires: The questionnaires listed in the “study endpoints” will be filled in at each weekly visit during each treatment period, but also at the first week following each treatment period: TP1_Week-1, TP1_Week-2, TP1_Week-3, TP1_Week-4, TP1_Week-5, TP1_Week-6, TP1_Week-7, TP1_Week-8, TP1_Week-9, TP1_Week-10, TP1_Week-11, TP1_Week-12 and Post-TP1_Week-1; followed by TP2_Week-1, TP2_Week-2, TP2_Week-3, TP2_Week-4, TP2_Week-5, TP2_Week-6, TP2_Week-7, TP2_Week-8, TP2_Week-8, TP2_Week-10, TP2_Week-11, TP2_Week-12, and Post-TP2_Week-1. A total of 26 times of data collection.

Biological samples: Biological tests listed in the “study endpoints” section will be performed by blood sampling on a monthly basis: TP1_Week-1; TP1_Week-5; TP1_Week-9; and Post-TP1_Week-1; and TP2_Week-1; TP2_Week-5; TP2_Week-9; and Post-TP2_Week-1. A total of 8 times of data collection.

### Biological Collection Procedure

The blood serum samples drawn for the need of the study will be transiently stored in the local biology department of each participating center during the study before being shipped to the coordinating center at the end of the study for analysis and long-term storage. The collection will be stored at −80°C at the coordinating center for a period of 10 years after the end of the study.

After completion of this study, stored samples may be used for further analysis, not described in the initial protocol but, which may be found useful in light of advances in scientific knowledge, provided the participant will be informed and will not oppose such use, as stated in the information note/consent form.

## Assessment and Measures

### Primary Endpoint

A response according to ASAS20 definition, assessed 1 week after the end of each treatment period is the primary endpoint of this study. ASAS20 response is defined as follows: an improvement of 20% compared to baseline and an absolute improvement from baseline of at least 1 unit, in 3 of the following 4 ASAS domains: patient’s global assessment, patient’s assessment of pain, BASFI, and inflammation (mean of the 2 morning stiffness-related BASDAI score questions), as well as no baseline deterioration of >20% and of at least one unit in the fourth domain.

### Secondary Endpoints

The secondary evaluation criteria for the study (collected at the end of each period) are:

1. Response according to ASAS40 definition, computed in a manner similar to that used to compute the ASAS20 response, except that it required improvement of 40% in (3 of the 4 ASAS domains, with a positive change of (2 units (on a scale of 0–10) in each domain. Absence of deterioration in the remaining domain is required and defined as for the ASAS20 response.
2. Partial remission according to the ASAS definition defined as a value of < 2 units (on a scale of 0–10) in each of the 4 ASAS domains (patient’s global assessment, pain, BASFI, and inflammation).
3. Change in BASFI.
4. Serum CRP level and ESR.
5. Change in ASDAS_CRP, ASDAS_ESR.
6. The level of IL-6, IL-17, IL-23, IL-33, and MMP-3-8-9.
7. SF-36, ASQOL‘.
8. ASAS-HI, WPI.
9. Fatigue (VAS), Global Pain (VAS).
10. HAD.
11. BASMI.
12. Change in NSAID intake score.

### Statistical Analysis

#### Calculation Hypotheses for the Number of Participants Required and the Result

Assuming a correlation coefficient of 0.1 for the within-patient response, 30% ASAS20 responders in the dummy stimulation period vs. 50% with active simulation, and the use of a random intercept logistic model for the statistical analysis using a 5% two-tailed threshold of significance, simulations have shown that a sample size of 90 subjects will provide 80% power for demonstrating the efficacy of active stimulation vs. placebo. Accounting for 25% potential missing values for the primary endpoint (loss to follow up, redrawn consent), we will include 120 patients in the study.

#### Sample Description

The characteristics of patients of each group will be described using descriptive statistics such as percentages for categorical data, means, medians, ranges and interquartile ranges for continuous variables. Groups of patients will also be described at the beginning of each period and of each treatment.

#### Statistical Analysis for the Primary Objective

The main statistical analysis will be performed on all randomized patients according to the Intention-To-Treat principle. A secondary analysis of the main criterion will be performed in the per protocol population comprised of patients having complied with at least 10 weeks of treatment during each treatment period, for whom all the other stipulations of the protocol have been respected and for whom all required data are available.

A subject-specific random intercept logistic model will be used to assess and test, using a 5% two-tailed threshold of significance, the effect of treatment on the percentage of ASAS20 response at the end of each period.

#### Statistical Analysis for the Secondary Objectives

Statistical analysis of secondary binary outcomes (i.e., response according to ASAS40 criteria and partial remission according to the ASAS definition) will be performed using similar random effect logistic models as for the primary objective. Statistical comparison between treatment periods of continuous variables (ASDAS_CRP, ASDAS_ESR, BASFI, BASMI, fatigue (BASDAI 1st question), global pain score, NSAID intake score scores, SF-36, ASQOL, ASAS HI and WPI scores, HAD, CRP serum level, ESR, levels of circulating IL-6, IL-23, IL-17, IL-33, and MMPs) will be performed using random effect linear models.

The analyses for the primary and secondary outcomes will be also performed in subgroups of patients (patients with radiographic forms of the disease and patients with non-radiographic forms of the disease), as sensitivity analyses.

All calculations will be performed using R software version 3.5.1 or later, using lme4 and lmerTest packages for the mixed effects models. Statistical tests will be performed using a Kenward-Roger method for degrees of freedom method estimation.

All statistical comparisons will be performed at the 5% level of significance.

No interim analysis is forecasted.

In case of missing data for the primary assessment criterion, no strategy of imputation is forecasted. We will take into account information available at time of last follow-up.

### Quality Control and Insurance

For this Minimal Risks and Burden research study, the appropriate quality control level has been determined based on the impact and the budget of the research. Assistance Publique-Hôpitaux de Paris (AP-HP) is the sponsor of this study and, by delegation, the DRCI (Clinical Research and Innovation Department) carries out the study’s missions in accordance with Article L.1121-1 of the French Public Health Code. Assistance Publique–Hôpitaux de Paris (AP-HP) has taken out insurance with HDI–GLOBAL SE through BIOMEDIC-INSURE for the full study period, which covers its own public liability and that of any collaborator (physician or research staff), in accordance with Article L.1121-10 of the Code de la Santé Publique (French Public Health Code). A Clinical Research Associate (CRA) appointed by the sponsor will be responsible for the good completion of the study, for collecting, documenting, recording and reporting all handwritten data, in accordance with the Standard Operating Procedures applied within the Clinical Research and Innovation Department. The investigator and the members of the investigator’s team agree to make themselves available during regular Quality Control visits carried out by the Clinical Research Associate.

## Conclusion

Current pharmacological management of inflammatory rheumatism and in particular axial SpA remains imperfect. Only 50% of patients respond to the most effective biotherapies, and many of them are only partially relieved (Braun and Sieper, 2002; Wendling and Prati, 2011). In addition, these are extremely expensive treatments that expose them to the risk of potentially serious side effects (de Koning et al., 2018; Lopez-Medina et al., 2018; Molto et al., 2018; Smolen et al., 2018; Wendling et al., 2018). The efficacy of non-invasive VNS therapy in patients with axial SpA has never been studied. This study will be the first to assess its benefit-risk balance through a rigorous randomized double-blind trial. This multi-center study includes 16 rheumatology centers in public hospitals in France, mostly university hospitals, all with good experience in conducting clinical trials. Our goal is to demonstrate the efficacy of non-invasive VNS as a treatment for axial SpA, especially in cases with resistance to biotherapies. We also expect improved quality of life and reduced short-term and long-term osteo-articular structural consequences in patients receiving VNS therapy. Proving efficacy of non-invasive VNS for reducing inflammation and its consequences in humans would open the way to clinical trials in other chronic inflammatory diseases.

## Co-investigators

Baillet Athan, CHU de Grenoble, Grenoble (ABaillet@chu-grenoble.fr); Emmanuelle Dernis, CH le Mans, Le Mans (edernis@ch-lemans.fr); Erick Legrand, CHU d’ Angers, Angers (ErLegrand@chu-angers.fr); Marie-Christophe Boissier, Hôpital Avicenne (AP-HP), Bobigny (Marie-christophe.boissier@aphp.fr); Christian Jorgesen, CHU de Montpellier, Montpellier (christian.jorgensen@inserm.fr); Daniel Wendling, CHU de Besançon, Besançon (dwendling@chu-besancon.fr); Thierry Lequerré, CHU de Rouen, Rouen (thierry.lequerre@chu-rouen.fr); Christian Marcelli, CHU de Caen, Caen (Marcelli-c@chu-caen.fr); Jacques-Eric Gottenberg, CHU de Strasbourg, Strasbourg (jacques-eric.gottenberg@chru-strasbourg.fr); Thao PHAM, Hôpital Sainte Marguerite, Marseille (Thao.PHAM@ap-hm.fr); Eric Lespessailles, CHR de Orléans, Orléans (eric.lespessailles@chr-orleans.fr); Philippe Goupille, CHU de Tours, Tours (philippe.goupille@univ-tours.fr); Jean-Marie Berthelot, CHU de Nantes, Nantes (jeanmarie.berthelot@chu-nantes.fr); Martin Soubrier, CHU Gabriel Montpied, Clermont-Ferrand (msoubrier@chu-clermontferrand.fr); and Grégoire Cormier, CHD Vendée, La Roche sur Yon (Greigoire.cormier@chd-vendee.fr).

## Ethics Statement

The trial has been approved by the Ethics Committee “Comité de protection des personnes du Sud-Ouest et Outre-Mer 4” (N°IRB: IORG0009855) on October 2nd 2020. The patients/participants provided their written informed consent to participate in this study.

## Author Contributions

EA and MB conceived the study. EA, GB, MB, LG, MJ, CL, MR, A-LR, FC, SC, and LN initiated the study design and helped with implementation. LG, MJ, and LN provided methodological and statistical expertise in clinical trial design. LN conducted the primary statistical analysis. All authors contributed to refinement of the study protocol and approved the final manuscript.

## Conflict of Interest

The authors declare that the research was conducted in the absence of any commercial or financial relationships that could be construed as a potential conflict of interest.

## Footnotes

1 http://www.spirit-statement.org/

## Abbreviations

Ach: Acetylcholine
ANSM: French National Agency for Medicines and Health Products Safety
AP-HP: Assistance Publique-Hôpitaux de Paris
ASAS20: Assessment in Ankylosing Spondylitis 20
ASAS HI: Health Index of patient with SpA
ASDAS: Ankylosing Spondylitis Disease Activity Score
ASQOL: Ankylosis Spondyloarthritis Quality of Life
BASDAI: Bath Ankylosing Spondylitis Disease Activity Index
BASFI: Bath Ankylosing Spondylitis Fonctional Index
BASMI: Bath Ankylosing Spondylitis Metrology Index
CAP: Cholinergic anti-inflammatory pathways
CHU: Centre Hospitalier Universitaire
CNIL: French Data Protection Agency
CPP: Comité de Protection des personnes
CRA: Clinical Research Associate
CRF: Case Report Form
CRP: C-reactive protein
CV: Curriculum Vitae
EKG: Electrocardiogram
ESR: Erythrocytes sedimentation rate
GDPR: General Data Protection Regulation
HAD: Hospital Anxiety and Depression scale
HLA-B27: Human Leucocyte Antigen B27
IL: interleukin
MMP: Matrix metallopeptidases
NSAID: Non-steroidal anti-inflammatory drugs
PHRC: Programme Hospitalier de Recherche Clinique
RA: Rheumatoid arthritis
SF-36: Short Form 36
SpA: SpondyloArthritis
TP: Treatment period 1
TNF: tumor necrosis factor
t-VNS: trans-cutaneous vagal nerve stimulation
VAS: Visuo Analogic Scale
VNS: Vagus nerve stimulation
WPI: Productivity at Work Index.
